# Evaluation of bone formation on orthopedic implant surfaces using an ex-vivo bone bioreactor system

**DOI:** 10.1038/s41598-021-02070-z

**Published:** 2021-11-18

**Authors:** Rupak Dua, Hugh Jones, Philip C. Noble

**Affiliations:** 1 Department of Chemical Engineering, School of Engineering and Technology, Hampton University, Hampton, VA USA; 2 Center for Orthopaedic Research, Innovation and Training, McGovern Medical School, UTHealth, Houston, TX USA

**Keywords:** Translational research, Biomedical engineering

## Abstract

Recent advances in materials and manufacturing processes have allowed the fabrication of intricate implant surfaces to facilitate bony attachment. However, refinement and evaluation of these new design strategies are hindered by the cost and complications of animal studies, particularly during early iterations in the development process. To address this problem, we have previously constructed and validated an ex-vivo bone bioreactor culture system that can maintain the viability of bone samples for an extended period ex-vivo. In this study, we investigated the mineralization of a titanium wire mesh scaffold under both static and dynamic culturing using our ex vivo bioreactor system. Thirty-six cancellous bone cores were harvested from bovine metatarsals at the time of slaughter and divided into five groups under the following conditions: Group 1) Isolated bone cores placed in static culture, Group 2) Unloaded bone cores placed in static culture in contact with a fiber-mesh metallic scaffold, Group 3) Bone cores placed in contact with a fiber-mesh metallic scaffold under the constant pressure of 150 kPa, Group 4) Bone core placed in contact with a fiber-mesh metallic scaffold and exposed to cyclic loading with continuous perfusion flow of media within the ex-vivo culture system and Group 5) Bone core evaluated on Day 0 to serve as a positive control for comparison with all other groups at weeks 4 and 7. Bone samples within Groups 1–4 were incubated for 4 and 7 weeks and then evaluated using histological examination (H&E) and the Live-Dead assay (Life Technologies). Matrix deposits on the metallic scaffolds were examined with scanning electron microscopy (SEM), while the chemical composition of the matrix was measured using energy-dispersive x-ray spectroscopy (EDX). We found that the viability of bone cores was maintained after seven weeks of loading in our ex vivo system. In addition, SEM images revealed crystallite-like structures on the dynamically loaded metal coupons (Group 4), corresponding to the initial stages of mineralization. EDX results further confirmed the presence of carbon at the interface and calcium phosphates in the matrix. We conclude that a bone bioreactor can be used as an alternate tool for in-vivo bone ingrowth studies of new implant surfaces or coatings.

## Introduction

The formation of a stable bone-implant interface is a prerequisite for the long-term success of any load-bearing implant^1–3^. Surface characteristics of the implant material, such as its chemical composition, morphology, and surface energy, all play a crucial role in successfully integrating the implant with the host bone^4^. The formation of new bone directly on the surface of metallic implants is mediated by the metabolic and secretory activities of bone cells, primarily osteoblasts^5^, and their interaction with the implant surface^6^. Through a series of steps, osteoblasts migrate from the native bone and form new bone tissue on the implant surface after initially depositing an organic non-collagenous matrix^7^, which then is seeded with nanocrystals of calcium phosphate, initiating mineralization. As these crystals grow, collagen assembly occurs through binding to the mineralized crystallites, and a layer of adherent bone tissue is created^5^.

Prior studies evaluating bony incorporation of implant surfaces have been primarily performed using animal models, most commonly the sheep^8^, dog^9,10^, pig^11^, or rabbit^12,13^. The recent emergence of new implant materials and manufacturing methods, including 3D printing, has greatly expanded opportunities for enhancing the biocompatibility and biomechanical performance of implantable devices. However, each new design, material and surface configuration must undergo testing to evaluate its biologic performance. Though this is theoretically possible using animal models, financial and ethical considerations make this approach prohibitive. To reduce the number of animals used for evaluation of new implant materials and surfaces while creating parsimonious solutions, alternative approaches to animal testing must be developed for prescreening.

While several bioreactors have been developed as an alternative to animal studies in bone biology and tissue engineering^14–17^, obstacles still persist through the incorporation of perfusion flow and mechanical loading for long-term culture. Historically, previous bioreactors lacked the capability of simultaneously providing programmable fluid flow, dynamic loading, and media exchange for an extended period of culture in an automated fashion. To overcome these deficiencies, we previously developed an *ex-vivo* organ culture system that provides the biochemical and mechanical environment necessary to maintain the viability of bone samples for 4 weeks with little maintenance^18^.

The objective of this study was to characterize the process of mineralization of an implant surface occurring in an ex-vivo bone bioreactor under a range of static and dynamic loading conditions. Preliminary studies were done to evaluate the validity of ex vivo bone bioreactor to sustain live bone for up to 7 weeks. Further, mineralization of the implant surface was assessed after incubation for 7 weeks under dynamic loading conditions.

## Material and methods

### Overview

An ex-vivo bone bioreactor that was previously developed and validated to keep the bone alive for 4 weeks was used to study the mineralization of an implant surface in an ex-vivo environment for 7 weeks and was compared with static cultures. Experimental time points were week 4 and week 7. In our study, we have 4 groups namely, Group 1) Isolated bone cores placed in static culture, Group 2) Unloaded bone cores placed in static culture in contact with a fiber-mesh metallic scaffold, Group 3) Bone cores placed in contact with a fiber-mesh metallic scaffold under the constant pressure of 150 kPa, Group 4) Bone core placed in contact with a fiber-mesh metallic scaffold and exposed to cyclic loading with continuous perfusion flow of media within the ex-vivo culture system. Outcomes for the bone coupons were assessed via live-dead assay, cell toxicity, and histology, while the outcomes for mineralization on the implant surfaces was assessed via SEM and EDX.

### Ex-vivo bioreactor system

**Figure 1:**
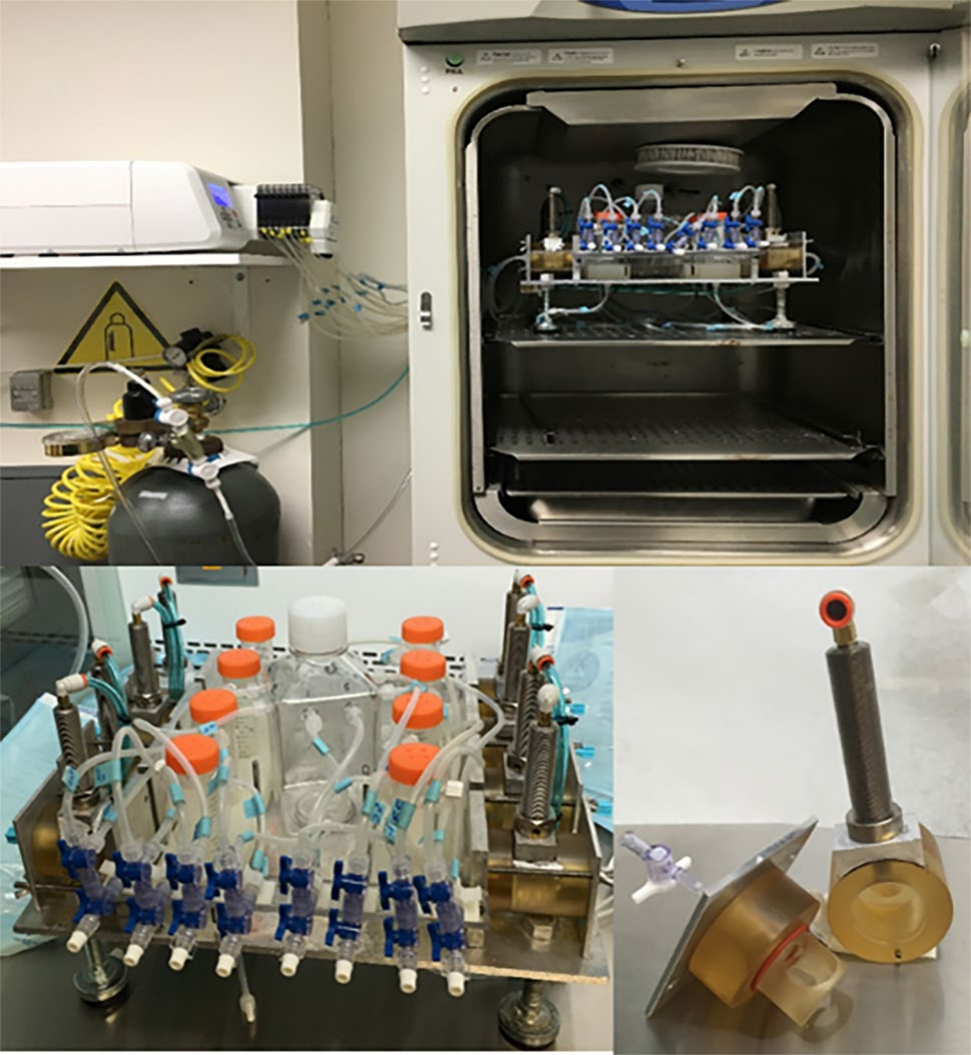
Ex-vivo bone bioreactor setup.

**Figure 2:**
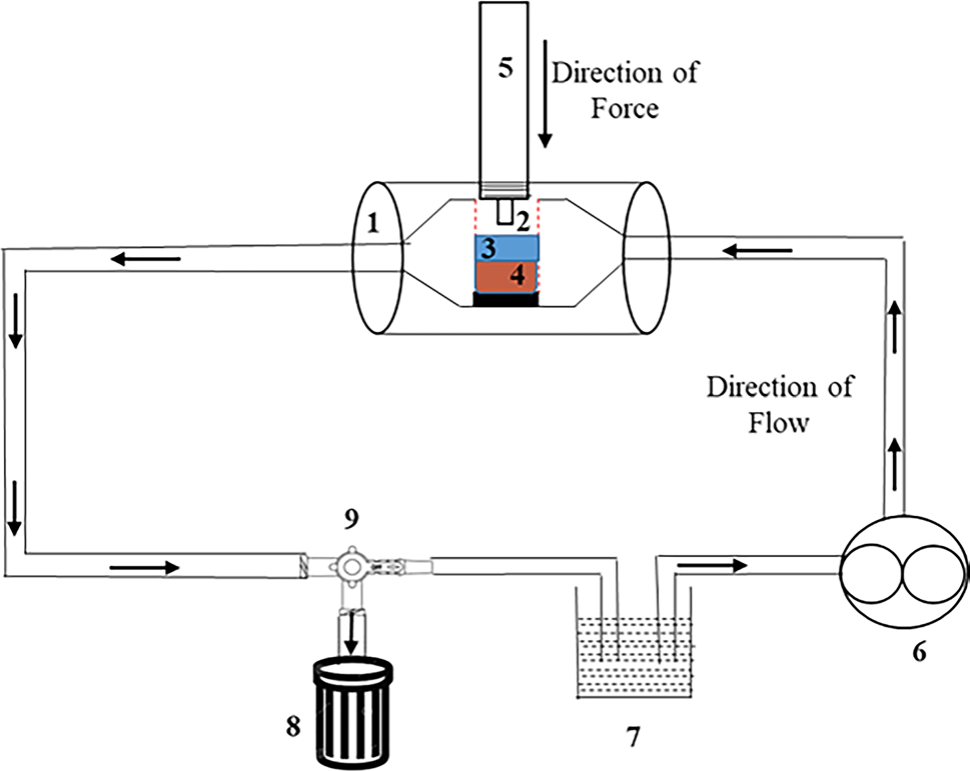
Schematic view of the setup of one complete specimen chamber in an ex-vivo bioreactor system. 1. Specimen chamber, 2. Polycarbonate holder, 3. Fiber Mesh metallic Scaffold, 4. Bone Core, 5. Pneumatic Actuator, 6. Peristaltic pump, 7. Media container, 8. Waste container, 9. Three-way valve.

We previously reported the performance of an ex-vivo bone bioreactor system that was shown to maintain viable bone for periods of up to 4 weeks^18^. This bioreactor system consisted of eight bivalved chambers mounted on an aluminum base plate (Fig. 1). Each chamber was made of polysulfone (McMaster-Carr, Atlanta, GA, USA), which is MRI, X-ray, and autoclave compatible. Internally, the ends of each specimen chamber are tapered at 60°–65° to promote laminar fluid flow and prevent any turbulence that may hamper cell growth. Schematic of the setup of one complete specimen along with the direction of flow and force from the actuator has been shown in Fig. 2. Each specimen chamber has a separate medium supply to avoid cross-contamination but one common collector for waste and used medium. Media exchange is performed by introducing sterile fresh media into individual vented media reservoirs (50 mL centrifuge tube, Fisher Scientific, Waltham, MA, USA) via hypodermic injection ports, which feed the medium to the specimen chamber through a multichannel peristaltic pump (Cole Parmer FH100M, Vernon Hills, IL, USA) via platinum cured silicon tubing (Masterflex tubing, Cole Parmer). Within the bioreactor, the medium is continuously circulated using an eight multichannel peristaltic pump. which can be programmed to provide flow rates from 0.002 to 760 mL/min per channel.

For our study, we set the flow rate at 1 mL/min because it has been found that the flow rate of 1 mL/min will not introduce the effective shear stress for overstimulating bone cells^18–20^. Further, each specimen chamber Is attached to a programmable pneumatic actuator to provide adjustable intermittent mechanical stimulus.

### Retrieval of bone cores

Thirty-six cylindrical (15 mm dia. × 10 mm) trabecular bone cores were harvested from bovine metatarsals in a sterile environment within 24 h of the sacrifice of the donor in an animal processing facility. The soft tissues were removed from the bone under sterile conditions. During machining, the bone was continuously irrigated with 0.9% NaCl solution at 4 °C to minimize heating and maintain the porosity of the native bone. After harvesting, the bone samples were immersed in a 5% antibiotic–antimycotic solution (Sigma Aldrich) consisted of penicillin, streptomycin and amphotericin B in Phosphate Buffered Saline (PBS) (Sigma Aldrich, St Louis, MO, SA). Each specimen was then washed twice with PBS at 37 °C, followed by PBS/5% antibiotic–antimycotic solution for another 10 min. Four bone cores in the positive control group (Group 5) underwent a live/dead assay and were later fixed in 10% formalin for histological examination. The remaining 32 cores were placed in 6 well plates in basal media with 10% FBS and 3% antibiotic–antimycotic solution supplemented with 5 mM β-glycerophosphate, 5 mg/L ascorbic acid, and 0.12 g/L sodium hydrogen carbonate. Each core was immersed for 24 h at 37 °C and 5% CO_2_ prior to the static and dynamic bioreactor experiments.

### The fiber-mesh metallic scaffold

After bone core processing, 24 fiber mesh titanium coupons of dimensions 1 cm × 1 cm × 0.5 cm (porosity: 50%; contact area: 0.15 in.^2^) were obtained by machined from the surface of cementless hip prostheses (Zimmer Inc., Warsaw, IN, USA). All coupons were washed in a soap solution and ultrasonicated with acetone and then ethanol for 10 min prior to sterilization by autoclave at a pressure of 17 psi for 30 min prior to use.

### Static culture of bone cores

After soaking in media for 24 h, 24 of the harvested bone cores were divided into three groups of eight and cultured under the following conditions: (1) Group 1—Bone core in static culture; (2) Group 2—Bone core in contact with a fiber-mesh metallic scaffold in static culture; (3) Group 3—Bone core with a fiber-mesh metallic scaffold under the constant pressure of 150 kPa in static culture. All samples for three different groups were cultured in 6-well plates. Every seven days, the media was collected and used for measuring pH then replaced. Four samples from each group were retrieved at week 4 and week 7 to evaluate viability using live dead assay (Life Technologies, USA) and assess the microscopic anatomy of cells using histology stain (H&E).

### Dynamic culture of bone cores in the ex-vivo bone bioreactor

Eight harvested cores were placed in the specimen chamber along with the fiber-mesh metallic scaffold in the ex-vivo bioreactor (Group 4). Each chamber was closed and placed in the mount on the aluminum base plate, and a pneumatic actuator was attached to each chamber. The whole bioreactor setup was placed in an incubator at 37 °C, and 5% CO_2_ and all necessary tubing connections were made. Each bone core was exposed to a continuous flow of nutrients. The actuator applied the cyclic mechanical loading of 9.6 lb_f_ (42.7 N) at a frequency of 1 Hz using the programmable pneumatic controller. Each specimen was loaded for 15 min every 8 h with 10 s recovery periods between cycles^18,21,22^. This loading regime provided both the short and long recovery periods necessary for bone cells to restore their mechanosensitivity. These values were based on our previous study^18^ and other in-vivo studies of the dynamic loading response of bone^21,22^. The fresh media was introduced through a syringe into a bioreactor through an inlet port and changed after every 7 days by opening the incubator. This change of media was done quickly so as not to affect the temperature and gas equilibrium with the bioreactor. During every medium change from each reservoir chamber, the used medium was collected for measuring pH by turning the 3-way valve. Four bone samples along with metallic scaffolds were also retrieved at both week 4 and week 7.

### Bone viability

A Live-Dead assay was performed to assess the viability of bone cores harvested at week 4 and week 7 by staining with a Calcein AM/Ethidium homodimer (Life Technologies) stain according to the manufacturer’s protocol. Each core was washed with PBS prior to staining and incubated for 30 min. Next, they were washed three times with PBS solution to remove the background fluorescence and subsequently visualized under the confocal fluorescent microscope (Olympus FV-1000, Olympus America Inc., Miami, FL, USA) at excitation and emission wavelengths of 468 nm and 568 nm, respectively^23^.

### Morphological assessment

The morphological changes of the bone coupons over 7 weeks were evaluated using H&E staining with the baseline bone core harvested at Day 0 acting as a positive control. Briefly, bone samples taken from the bioreactor and from the static loading groups after week 4 and week 7 were fixed in 10% formalin and then decalcified. The decalcified bone cores were embedded in paraffin, sectioned to 10 µm with a microtome, and stained with hematoxylin and eosin (H&E) according to the manufacturer’s protocol (ScyTeck Laboratories, UT, USA). Each section was viewed under a bright field microscope at random locations to visualize and compare the morphology with all the group specimens^24^.

### Cell toxicity

The toxicity of the bone cores was evaluated by measuring the change in the pH of the medium every week for 7 weeks. The change in pH is an indication of the difference in the microenvironment of the bone. The acidification shows an association with the onset of the apoptosis of the cells in culture^25–27^. Briefly, 3 mL of used media was collected from the bioreactor group and static samples groups at week 1 to week 7 at the time of media exchange. pH was measured using a pH meter and recorded and analyzed.

### Scanning electron microscopy (SEM)

The metallic fiber-mesh scaffolds for different time points (week 4 and week 7) for all groups (Groups 2, 3 and 4) were observed under SEM for indications of mineralization and tissue incorporation. Briefly, the samples were first fixed in 2.5% glutaraldehyde for 30 min and then washed twice in media. The specimens were then dehydrated in a graded series of ethanol (20%, 50%, 70%, 95%, 100%) and then in a 1:1 mixture of 100% ethanol and t-butanol alcohol and left to air dry overnight. Samples were then sputter-coated with platinum (coating thickness: 7 nm) and examined under a Nova NanoSEM 230 (FEI, Oregon, USA) high-resolution field emission scanning electron microscope to provide representative SEM images for each group.

### Energy-dispersive X-ray spectroscopy (EDX)

The samples used for SEM were further analyzed analytically for the elemental content of the matrix at the interface between the bone and the implant using energy-dispersive X-ray spectroscopy (EDX) detector (EDAX Inc., Mahwah, NJ, USA) that was added on the Nova NanoSEM 230 system.

### Statistics

All data are expressed as average ± standard error. Statistical analyses of the results obtained the pH results were performed using commercially available software (SPSS, IBM, version 27, Armonk, NY, USA). A one-way ANOVA and post hoc Tukey test was used to compare means and to determine statistically significant differences (*p* < 0.05) between groups, respectively^28,29^.

## Results

### Cell viability

**Figure 3:**
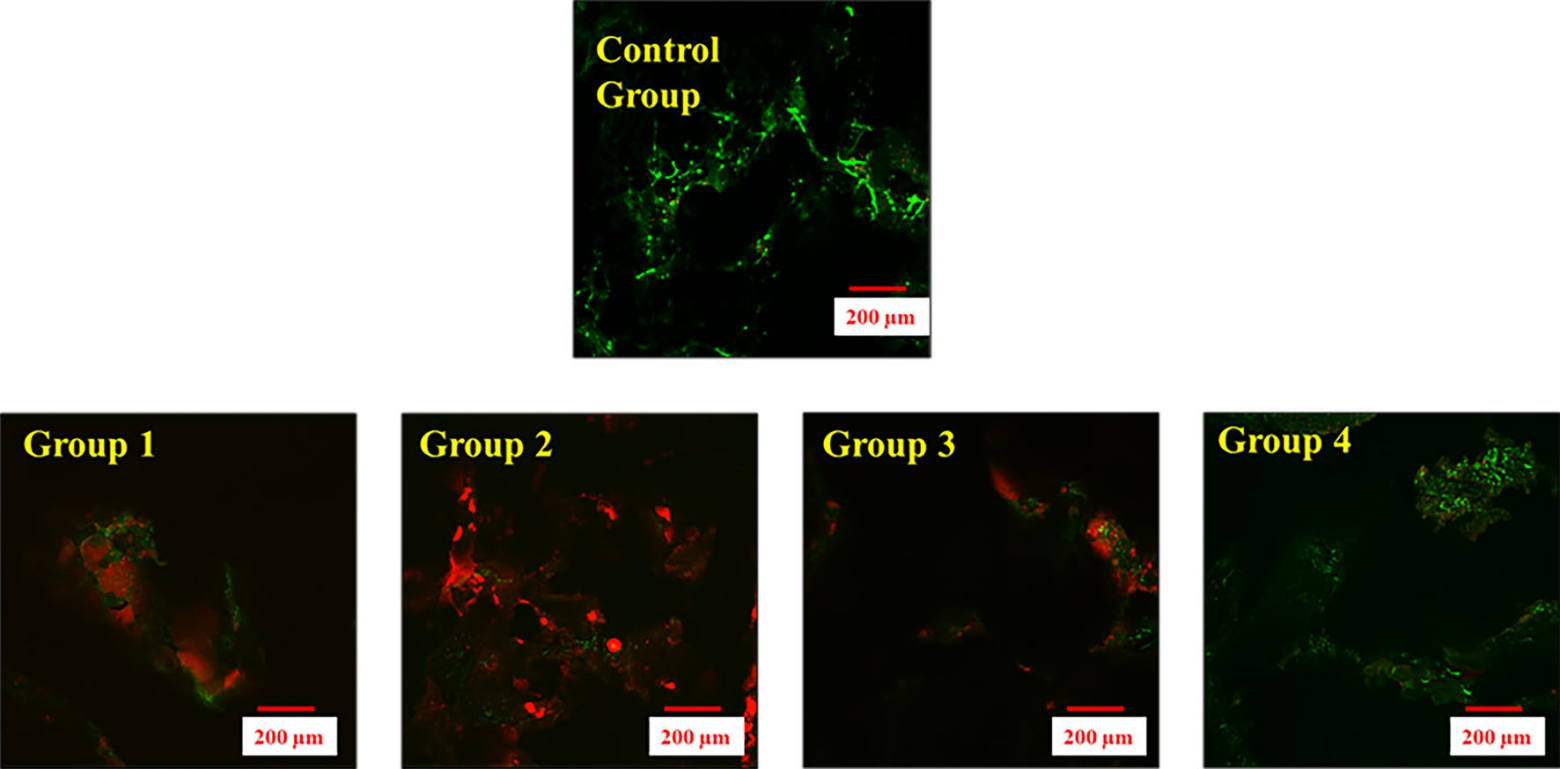
Live Dead Assay of bone coupons for different groups at week 7. Group 1) Isolated bone cores placed in static culture, Group 2) Unloaded bone cores placed in static culture in contact with a fiber-mesh metallic scaffold, Group 3) Bone cores placed in contact with a fiber-mesh metallic scaffold under the constant pressure of 150 kPa, Group 4) Bone core placed in contact with a fiber-mesh metallic scaffold and exposed to cyclic loading with continuous perfusion flow of media within the ex-vivo culture system. Green color indicates the live cells while red color indicates the dead cells. Live cells, similar to control group (Positive Control Bone Core at Day 0) were observed in Group 4 while dead cells were observed in Groups (1–3).

The representative images of the viability of the bone cells in all the groups maintained for 7 weeks revealed that the live cells (green color) in the bone cores that were retrieved from the bioreactor at both week 4 and week 7 similar to the results obtained for Day 0 bone core, which acted as a positive control. The bone cores that were retrieved from the static culture groups (Group 1–3) showed more dead cells (red color) at week 4 and at week 7. Only week 7 data have been shown (Fig. 3).

### Morphological assessment

**Figure 4:**
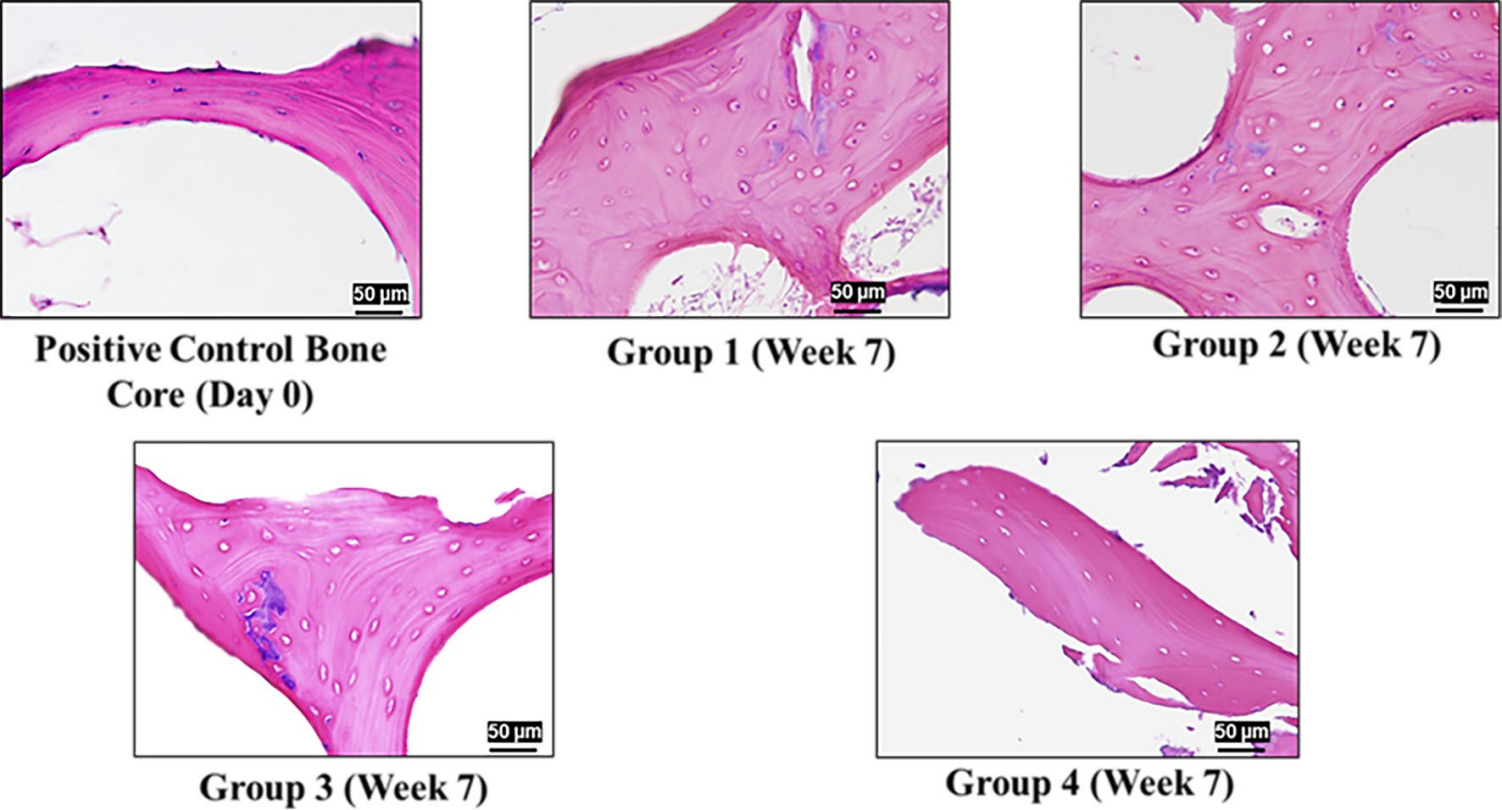
H&E Staining for different groups bone cores at week 7. Group 1) Isolated bone cores placed in static culture, Group 2) Unloaded bone cores placed in static culture in contact with a fiber-mesh metallic scaffold, Group 3) Bone cores placed in contact with a fiber-mesh metallic scaffold under the constant pressure of 150 kPa, Group 4) Bone core placed in contact with a fiber-mesh metallic scaffold and exposed to cyclic loading with continuous perfusion flow of media within the ex-vivo culture system. Cells in group 4 maintained a similar cell structure similar to one in control, while cells in Groups 1–3 changed to a round-like shape.

Histology images at 20× objective magnification revealed that, in the Bioreactor group, the elongated shape of the cells at Day 0 was retained after 4 weeks and even at week 7 (Fig. 3). However, in other groups at week 4 and week 7 weeks, the cells remodeled themselves, were more round in shape, and they retained this round shape till week 7 (Fig. 4).

### Cell toxicity

Over a period of 7 weeks, the pH value remained consistent for the bioreactor group samples, but there was a progressive decrease in the pH in all other static groups at every test point (Table 1). By week 4, the pH value had dropped below 7 for all static groups (Groups 1–3) in comparison with 7.19 ± 0.01 for the bioreactor group. As the incubation period increased, there was a statistically significant decline in the pH value for the static group compared with the pH values in the bioreactor group (Group 4). The pH of the medium (control) was 7.32 ± 0.02.

**Table 1:** Measurement of pH in the media (n = 4) during incubation of bone cores over 7 weeks for four groups, means ± SE.

Time point of sampling	Group 1	Group 2	Group 3	Group 4
Week 1	7.22 ± 0.041	7.26 ± 0.033	7.23 ± 0.016	7.23 ± 0.009
Week 2	6.98 ± 0.017	7.03 ± 0.009	7.03 ± 0.010	7.23 ± 0.006*
Week 3	6.91 ± 0.028	7.04 ± 0.003*	7.03 ± 0.007*	7.21 ± 0.008*^^˜^
Week 4	6.86 ± 0.030	6.98 ± 0.008*	6.98 ± 0.024*	7.19 ± 0.005*^^˜^
Week 5	6.82 ± 0.028	6.98 ± 0.009*	6.93 ± 0.006*	7.22 ± 0.010*^^˜^
Week 6	6.72 ± 0.029	6.90 ± 0.022*	6.90 ± 0.043*	7.22 ± 0.010*^^˜^
Week 7	6.67 ± 0.023	6.89 ± 0.011*	6.86 ± 0.012*	7.22 ± 0.006*^^˜^

“*” indicates that the difference between the group compared with Group 1 was significant (*p* < 0.05), “^” indicates that the difference between the group compared with Group 2 was significant (*p* < 0.05) and “˜” indicates that the difference between the group compared with Group 3 was significant (*p* < 0.05).

Group 1—Bone core in static culture; Group 2—Bone core in contact with a fiber-mesh metallic scaffold in static culture; Group 3—Bone core with a fiber-mesh metallic scaffold under a pressure of 150 kPa in static culture; Group 4: Bone core in Dynamic culture in an ex-vivo bone bioreactor.

### SEM

**Figure 5:**
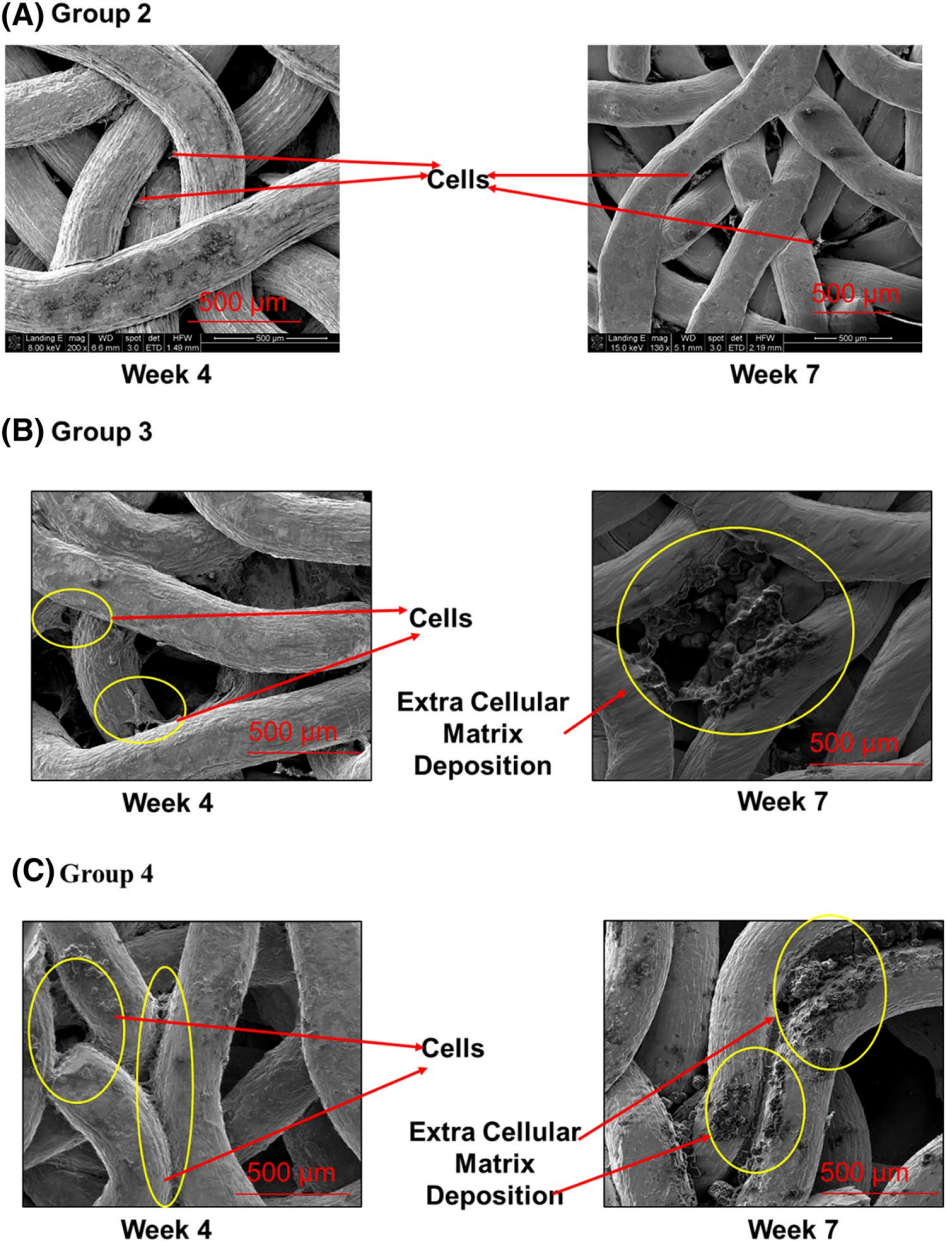
SEM pictures showing the progress of new bone tissue formation from week 4 to week 7 on the metallic scaffold for Groups 2, 3, and 4. (**A**) Fiber-mesh metallic scaffold in contact with bone core in static culture (**B**) Fiber-mesh metallic scaffold in contact with bone under a pressure of 150 kPa in static culture (**C**) Fiber-mesh metallic scaffold in contact with bone core and exposed to cyclic loading with continuous perfusion flow of media within the ex-vivo culture system.

**Figure 6:**
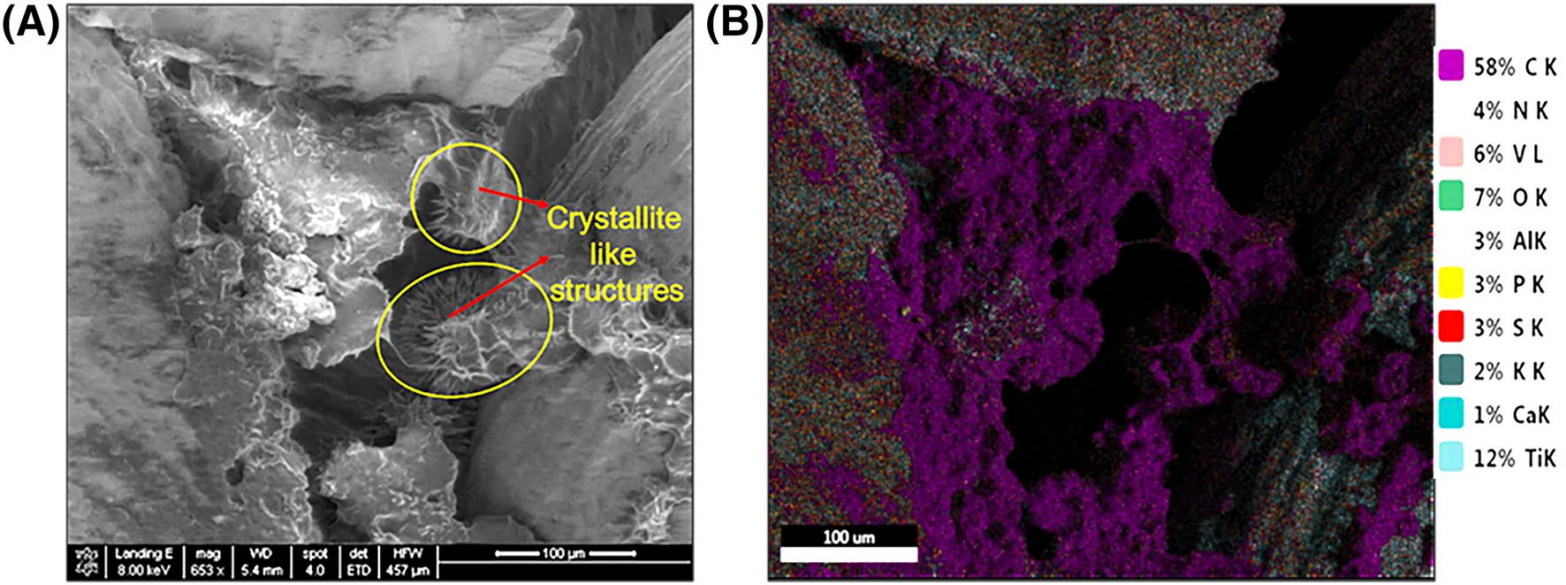
SEM (Left) and EDX (Right) image of wire mesh scaffold in contact with the bone pulled out from bioreactor at week 7 at 653× magnification. SEM image showing the presence of crystallite like structures and EDX showing the presence of elements present on the matrix at the same location shown in the SEM image.

SEM images for Group 2, in which the titanium implant was in contact with the bone core in static culture, showed that there was little cell attachment to the metal fibers at either time point and minimal change in cell attachment from week 4 to week 7 (Fig. 5A). SEM images of Group 3, (static interface loading) showed more cell attachment than the Group 2 samples at week 4, and some areas of deposition of extracellular matrix at week 7 (Fig. 5B). Cell attachment was markedly increased at both 4 and 7 weeks in fiber-mesh metallic scaffolds that had been loaded in the bioreactor (Group 4). In these specimens, all titanium fibers were covered with extracellular matrix (Fig. 5C), and at 7 weeks, crystallite-like structures were visible in areas within the organic matrix (Fig. 6a).

### EDX

EDX results confirmed the presence of an organic matrix deposited on the scaffold and was made up of mostly carbon. Figure 6a showing the SEM image of the week 7 wire mesh sample that was retrieved from the bioreactor, and Fig. 6b, showing the elementary analysis of the same image. We observed the presence of carbon in between the gaps of the fiber mesh metal and also on the top of metal, as depicted by the pink shaded areas within the figure. Chemical analysis also revealed the presence of calcium and phosphates on the metal samples.

## Discussion

We found that an implant material cultured with a live ex-vivo bone in a bioreactor accurately simulated the processes reported during bio-incorporation of metallic implants in vivo^30–32^. Our results demonstrate that our ex-vivo bone bioreactor is capable of keeping the bone alive in an ex-vivo environment for extended periods of up to 7 weeks. This is sufficient time for bone formation in animal models^30,31^. This long-term culture of bone ex-vivo was previously limited to around 4 weeks by other systems that were developed to culture ex-vivo bone^14^. We also reproduced the initial events leading to osseous incorporation of implants in vivo, which entails migration of bone cells to the metallic surface, deposition of an extracellular matrix and formation of calcium-rich crystallites^33^. In one previous study, Kajiwara et al., found that the amount of new bone formed on titanium implants in-vivo increased with the duration of implantation^31^. Though we didn’t quantify the bone formation in our study, we observed a similar trend with time in the extracellular matrix formation in our bioreactor group.

From Live-Dead fluorescent images, we observed that the bone cores cultured in the bioreactor group had more viable cells similar to those found in the positive control, indicating that the ex-vivo bone bioreactor is capable of keeping the bone viable for periods of up to 7 weeks. In contrast, the bone cores cultured under static conditions, we found more red color in the live dead images (Fig. 2), indicating the bone cores were not viable. This was further confirmed by the results of H&E staining at 4 and 7 weeks that revealed the remodeling of bone cells in the three static groups from an elongated to a round shape at 4 weeks, and the presence of empty lacunae. It is postulated that these changes were primarily due to the increase in the acidity of the medium culture in the static groups. In contrast, in the bone cores cultured in the ex-vivo bioreactor the elongated morphology of cells seen observed in the positive control was maintained at 7 weeks.

We found a slight decrease in the pH value for all the groups when compared to the pH of the control medium (7.32 ± 0.05), indicating the production of lactic acid from cellular metabolism (Reference) for all the groups (Table 1). Over time, the pH value dropped significantly and became more acidic in the static culture groups which is consistent with the loss of cellular viability through apoptosis. In contrast, the pH of the media in the bioreactor group remained stable in the range of 7.19–7.22, indicating cell growth and viability, as confirmed by Live-Dead staining. It has been found that in static culture, the transfer of nutrients does not take place, so it leads to the death of tissue in the center, affecting the viability of the cells^34^. Our results were consistent with the earlier investigation where they observe dead cells in the static medic where there is no flow^35^.

Once we validated that the bone was kept alive for up to 7 weeks in the ex-vivo bone bioreactor, we examined the extracellular deposition on the metallic wire mesh scaffolds for groups (1–4). SEM images revealed extensive cell attachment and colonization on the metal scaffolds pulled from the bioreactor group at week 4 in contrast to little and no attachment in the static group. This may be because the bone cores were still viable in group 4, promoting the migration of bone cells to the scaffold. After 7 weeks of culture in a dynamic environment, SEM images showed the presence of globular matrix formation at the interface between the bone core and the fiber mesh of the metallic implant. These results were consistent with previous in-vivo^32^ and in-vitro studies^7^ where they observed migration of cells from bone to the interface surface. In contrast, we found little extracellular matrix deposition on samples under pressure in static culture and no extracellular matrix deposition on the unweighted samples in a static culture in week 7. This may be due to the presence of non-viable bone cores, as indicated from the cell viability and histology results.

We also observed the formation of nanocrystals on the surface of the metal’s samples obtained from the bioreactor group at week 7 (Fig. 5a), showing the process of mineralization taking place. Thus, the initial events that occur during the formation of new bone^5^ were confirmed by the SEM images of the samples retrieved from the bioreactor.

Further EDX results confirmed the presence of organic matrix rich in carbon on the scaffolds obtained from the bioreactor group. We also found the presence of calcium phosphate on the metal samples. These are the initial events for the formation of bone in-vivo, and we were able to demonstrate these events successfully in an *ex-vivo* bone reactor system.

These promising results support the conclusion that this novel bone ex-vivo bioreactor has the potential to serve as an alternative to animal studies for screening new surface designs and implant coatings to assess their ability to promote osseointegration. However, we did not find strong integration between the bone core and the metallic scaffold at 7 weeks, though we suspect this will occur with more time in culture through a progression of mineralization of the collagen layer coating the metallic coupon. It has also been found that the bone in-growth on metallic implants in dog models starts at week 2, which we observed at week 7 in our bioreactor group and by week 12, ingrown bone began remodeling in dog models^36^. This implies that our bioreactor simulates the *in-vivo* environment but displays at a slower rate of osseointegration of metallic surfaces. This may be due to the fact that we are missing many natural biological events that happen simultaneously in the native environment during bone wound healing and new bone formation.

An additional limitation of this study was the lack of quantification related to cell viability and histology analysis. The porous nature of the trabecular bone that we used in our study limited our ability to obtain quantitative information without access to confocal imaging capabilities. Another limitation of the study was that we didn’t have the gender and age of the bone coupons used, and we used bovine bones that came from the slaughterhouse, which may have a different osteoblastic response when compared with human bones. However, this was not a major concern as the objective of the present study was to evaluate the feasibility of using an ex-vivo bioreactor as an alternative to animal studies in assessing the affinity of implant for supporting osseointegration.

Nonetheless, it is clear that the described ex-vivo bone culturing bioreactor system will potentially offer a means to empirically test implantable orthopedic devices during the design process in a cost-effective manner.

## Conclusions

In summary, we were able to demonstrate the validity of the bioreactor for keeping the bone viable for up to 7 weeks. We also showed the utility of ex-vivo bone reactor in bone ingrowth-related studies on implant surfaces. This system can serve as an indispensable tool in studying and developing orthopedic devices requiring fixation through ingrowth.
